# A phase Ib, open-label, dose-escalation trial of the anti-CD37 monoclonal antibody, BI 836826, in combination with gemcitabine and oxaliplatin in patients with relapsed/refractory diffuse large B-cell lymphoma

**DOI:** 10.1007/s10637-020-01054-6

**Published:** 2021-02-01

**Authors:** Monica Balzarotti, Massimo Magagnoli, Miguel Ángel Canales, Paolo Corradini, Carlos Grande, Juan-Manuel Sancho, Francesco Zaja, Anne-Marie Quinson, Valérie Belsack, Daniela Maier, Carmelo Carlo-Stella

**Affiliations:** 1 Oncology and Hematology Unit, Humanitas Cancer Center, Humanitas Clinical and Research Center – IRCCS, Rozzano, Milano, Italy; 2 Servicio de Hematologia, Hospital Universitario La Paz, Madrid, Spain; 3 University of Milan, Milan, Italy; 4 Division of Hematology, Fondazione IRCCS Istituto Nazionale dei Tumori, Milan, Italy; 5 Hospital Universitario 12 de Octubre, Madrid, Spain; 6 Clinical Hematology Department, ICO-IJC-Hospital Germans Trias i Pujol, Badalona, Spain; 7 University of Trieste, Ospedale Maggiore, Piazza dell’Ospitale 1, Trieste, Italy; 8 Boehringer Ingelheim Pharmaceuticals, Inc, Ridgefield, CT USA; 9 SCS Boehringer Ingelheim Comm.V, Brussels, Belgium; 10 Boehringer Ingelheim Pharma GmbH & Co. KG, Biberach an der Riß, Germany; 11 Department of Biomedical Sciences, Humanitas University, Milan, Italy

**Keywords:** BI 836826, CD37, Diffuse large B-cell lymphoma, Non-Hodgkin lymphoma, Phase I, Relapsed

## Abstract

*Background* BI 836826 is a chimeric mouse–human monoclonal antibody directed against human CD37, a transmembrane protein expressed on mature B lymphocytes. This open-label, phase I dose-escalation trial (NCT02624492) was conducted to determine the maximum tolerated dose (MTD), safety/tolerability, and preliminary efficacy of BI 836826 in combination with gemcitabine and oxaliplatin in patients with relapsed/refractory diffuse large B-cell lymphoma (DLBCL). *Methods* Eligible patients received intravenous infusions of BI 836826 on day 8 and gemcitabine 1000 mg/m^2^ plus oxaliplatin 100 mg/m^2^ on day 1, for up to six 14-day treatment cycles. Dose escalation followed the standard 3 + 3 design. *Results* Of 21 treated patients, 17 had relapsed/refractory DLBCL and four had follicular lymphoma transformed to DLBCL. BI 836826 dosing started at 25 mg and proceeded through 50 mg and 100 mg. Two dose-limiting toxicities (DLTs) occurred during cycle 1, both grade 4 thrombocytopenia lasting > 7 days, affecting 1/6 evaluable patients (17%) in both the 50 mg and 100 mg cohorts. Due to early termination of the study, the MTD was not determined. The most common adverse events related to BI 836826 treatment were neutropenia (52%), thrombocytopenia (48%), and anemia (48%). Eight patients (38%) experienced BI 836826-related infusion-related reactions (two grade 3). Overall objective response rate was 38%, including two patients (10%) with complete remission and six patients (29%) with partial remission. *Conclusions* BI 836826 in combination with GemOx was generally well tolerated but did not exceed the MTD at doses up to 100 mg given every 14 days.

**Supplementary Information:**

The online version contains supplementary material available at 10.1007/s10637-020-01054-6.

## Introduction

Diffuse large B-cell lymphoma (DLBCL) is the most common form of non-Hodgkin lymphoma (NHL) and accounts for approximately 30–40% of new NHL cases [1, 2]. The current standard of care for front-line treatment of DLBCL is immunochemotherapy: rituximab plus the multi-agent chemotherapy cyclophosphamide, doxorubicin, vincristine, and prednisone (R-CHOP) [3]. Up to 40% of patients relapse or become refractory to R-CHOP. However, despite years of intensive research into alternative front-line treatments, R-CHOP or R-CHOP-like therapies remain the standard of care. Poor response rates to front-line treatment have been associated with various prognostic factors, including clinical characteristic indices (International Prognosis Index [IPI], Revised-IPI [R-IPI], and NCCN-IPI) [4–6], high expression of certain tumor markers (e.g. β2-microglobulin), molecular rearrangements of oncogenes (e.g. *MYC*, *BCL2*), or cell of origin classification (e.g. activated B-cell-like origin) [7, 8]. Patients who are refractory to, or relapse after, first-line immunochemotherapy generally receive high dose chemotherapy followed by autologous stem cell transplant (ASCT) [9, 10]. However, outcomes following salvage treatment are generally poor. For instance, in the retrospective SCHOLAR-1 study of 636 patients with refractory DLBCL, the response rate to subsequent therapy was only 26% with median overall survival (OS) of 6.3 months [11]. Moreover, anti-CD20 based immunochemotherapy regimens appear to have low efficacy in the salvage setting. In the phase III CORAL trial, 3-year event-free survival in patients with relapsed/refractory DLBCL treated with R-ICE (rituximab, ifosfamide, carboplatin, etoposide) or R-DHAP (rituximab, dexamethasone, cytarabine, cisplatin) was 37% overall and only 21% in patients previously treated with rituximab [12]. Only 50% of patients could proceed to ASCT [13]. Thus, more effective salvage strategies for patients with relapsed or refractory disease after front-line immunochemotherapy are needed [2].

CD37 is a tetraspanin protein that is highly expressed on mature B lymphocytes and lymphoid neoplasms [14–16]. It appears to have multiple physiological functions, including regulation of apoptosis/survival signaling [17], B/T cell interaction [18], and T-cell proliferation [19]. Early clinical studies of anti-CD37 agents [20–22] have demonstrated that it is a viable therapeutic target in B cell NHL.

BI 836826 is a chimeric Fc-engineered immunoglobulin G1 (IgG1) monoclonal antibody directed against human CD37 [23]. In transgenic mice and cynomolgous monkeys, BI 836826 dose-dependently depleted peripheral B cells, and significantly suppressed tumor growth in a mouse B-cell lymphoma xenograft model [23]. In phase I studies, BI 836826 demonstrated acceptable tolerability and anti-tumor activity in patients with relapsed/refractory chronic lymphocytic leukemia (CLL) [24] and in patients with relapsed/refractory B-cell NHL [25]. As gemcitabine and oxaliplatin (GemOx), with or without rituximab, is a treatment option in patients with ASCT-ineligible relapsed/refractory DLBCL [26, 27], we investigated the feasibility of combining BI 836826 and GemOx in this phase I, dose escalation study.

## Patients and methods

### Patients

Eligible patients were aged 18 years or older and had histologically confirmed relapsed/refractory DLBCL (including transformed follicular lymphoma). ‘Relapsed’ was defined as the appearance of any new lesion after attainment of an initial complete response (CR) and ‘refractory’ was defined as < 50% decrease in lesion size with prior anti-lymphoma treatment in the absence of new lesion development. All patients had previously been treated with an anti-CD20 monoclonal antibody in combination with anthracycline-containing chemotherapy and were either ineligible for high dose therapy and ASCT or had relapsed/progressed after an autologous/allogeneic stem cell transplant. Allogeneic stem cell transplant performed at least 6 months prior to study entry was permitted if patients did not require immunosuppressive treatment and had no evidence of active graft-versus-host disease. Patients were required to have Eastern Cooperative Oncology Group (ECOG) performance status of 0–2, acceptable organ function, and a screening CT scan with involvement of at least one bi-dimensional lesion/node of > 1.5 cm.

Key exclusion criteria were: prior history of malignancy other than DLBCL; known central nervous system involvement; and receipt of anti-lymphoma treatment either within 14 days or, in the case of an investigational agent, less than five half-lives of the investigational drug, prior to the first dose of the current trial medication.

The study was conducted in accordance with the Declaration of Helsinki and Good Clinical Practice guidelines, and the study protocol was approved by the Institutional Review Boards or Independent Ethics Committees of all participating institutions. Written informed consent was obtained from all patients.

### Study design and treatment

This was an open-label, multicenter phase Ib dose-escalation trial conducted to determine the maximum tolerated dose (MTD), safety/tolerability, and preliminary efficacy of BI 836826 in combination with GemOx in patients with relapsed/refractory DLBCL (NCT02624492). BI 836826 (starting dose of 25 mg) was administered as an intravenous infusion on day 8 of each 14-day cycle, while gemcitabine (1000 mg/m^2^) and oxaliplatin (100 mg/m^2^) were given intravenously on day 1 of each cycle. Patients received a maximum of six treatment cycles; each cycle could be delayed by up to 14 days in cases of toxicity.

Paracetamol, antihistamine and glucocorticoid premedication to mitigate infusion-related reactions (IRRs) was administered 30–120 minutes prior to all BI 836826 infusions. Supportive care including blood products and growth factors were permitted according to local guidelines; prophylactic anti-infective agents were also allowed. Dose escalation was conducted according to the standard 3 + 3 design and was planned to continue until the MTD of BI 836926 in combination with GemOx was reached. BI 836826 dose increments of 50, 100, 150 and 200 mg were planned.

### Study assessments

The primary endpoints were the MTD and the number of patients with dose-limiting toxicities (DLTs) during the MTD evaluation period (cycle 1). The MTD was defined as the highest dose for which the number of patients with DLTs was no more than 17% during the MTD evaluation period (cycle 1).

DLTs included both non-hematologic and hematologic adverse events (AEs) considered related to any trial medication. This included any non-hematologic grade ≥ 3 AEs except for: laboratory abnormalities that could be corrected with treatment within 48 h; nausea, vomiting, or diarrhea that resolved within 48 h with adequate treatment; neuropathy considered related to oxaliplatin; or IRRs. Hematologic DLTs were: grade 4 neutropenia lasting > 7 days despite growth factor support; any febrile neutropenia that did not resolve within 48 h with appropriate treatment; grade 4 thrombocytopenia lasting > 7 days or grade 3/4 thrombocytopenia with clinically significant bleeding; failure to recover platelets ≥ 75 × 10^9^/L by 4 weeks after the start of the cycle; and failure to recover neutrophils ≥ 1.0 × 10^9^/L by 4 weeks after start of the cycle. Other safety assessments included the incidence and severity of AEs graded according to the Common Toxicity Criteria for Adverse Events (CTCAE, version 4.0) and changes in laboratory parameters.

The secondary endpoint was investigator-assessed best overall response according to International Working Group criteria for non-Hodgkin lymphoma [28] with refined evaluation of the PET scan using the 5-point scale [29].Tumor size reduction was analyzed as best percentage change from baseline in the sum of the product of the longest perpendicular diameters (SPD) based on imaging data. Pharmacokinetic analysis was planned but was not conducted.

### Statistical methods

No formal statistical testing was undertaken. The number of patients with DLTs and the best overall response was summarized by descriptive statistics.

## Results

### Patients and treatment exposure

Thirty-five patients were enrolled and 21 treated at seven centers in Belgium, Italy and Spain between March 10, 2016 and March 16, 2018. All patients were white and 48% were male (Table 1). Median age was 54.0 years (range 22 to 86), and 57%, 33% and 10% of patients had a baseline ECOG performance score of 0, 1 and 2, respectively. Of the 21 patients, 17 (81%) had pure DLBCL and four (19%) had follicular lymphoma transformed to DLBCL. Lymphoma was considered relapsed for 33% of patients, refractory for 38%, and progressive for 29%. All patients had previously received systemic therapy, with most having received one, two, or four previous therapies (33%, 29%, and 29% of patients, respectively); 71% had progressed following the last therapy.

**Table 1:** Patient demographics

	BI 836826 dose^a^			
	25 mg (*n *= 5)	50 mg (*n *= 8)	100 mg (*n *= 8)	Total (*N* = 21)
Male, *n* (%)	1 (20.0)	4 (50.0)	5 (62.5)	10 (47.6)
Race, *n* (%)				
White	5 (100.0)	8 (100.0)	8 (100.0)	21 (100.0)
Median age, years (range)	77.0 (22–86)	51.0 (41–78)	53.5 (42–77)	54.0 (22–86)
ECOG PS at baseline, *n* (%)				
0	5 (100.0)	4 (50.0)	3 (37.5)	12 (57.1)
1	0	2 (25.0)	5 (62.5)	7 (33.3)
2	0	2 (25.0)	0	2 (9.5)
Ann Arbor stage at screening, *n* (%)				
II	0	1 (12.5)	0	1 (4.8)
III	0	2 (25.0)	3 (37.5)	5 (23.8)
IV	5 (100.0)	5 (62.5)	5 (62.5)	15 (71.4)
Mean time from first diagnosis, years (SD)	4.4 (3.8)	5.1 (7.5)	2.9 (3.7)	4.1 (5.4)
Median number of prior therapies (range)	2.0 (1–4)	2.0 (1–4)	3.0 (1–6)	2.0 (1–6)
Prior therapy, *n* (%)				
Radiotherapy	2 (40.0)	1 (12.5)	1 (12.5)	4 (19.0)
Stem cell transplant	2 (40.0)	2 (25.0)	3 (37.5)	7 (33.3)
Surgery	0	2 (25.0)	1 (12.5)	3 (14.3)
Progression since last systemic therapy, *n* (%)	5 (100.0)	7 (87.5)	3 (37.5)	15 (71.4)

^a^Given every 14 days in combination with gemcitabine 1000 mg/m^2^ and oxaliplatin 100 mg/m^2^

*ECOG PS* Eastern Cooperative Oncology Group Performance Status, *SD* standard deviation

The best response to previous therapy was CR in five patients (23.8%), partial remission (PR) in two patients (9.5%), and stable disease (SD) in two patients (9.5%); 10 patients (47.6%) had progressive disease (PD); the status of two patients (9.5%) was unknown or missing. Important protocol deviations were noted for seven patients, three of whom had more than one protocol deviation: entrance criteria were not met for 6 patients (efficacy-related criteria for four patients, trial diagnosis for one patient, and safety criteria for one patient), administration of trial medication was delayed for three patients, and prohibited medication was administered for one patient (Supplementary Table 1).

All 21 patients discontinued the trial, with10 patients (48%) completing the maximum of six treatment cycles. Of the 11 patients who discontinued before completing six cycles, six (29%) discontinued due to AEs, and 5 (24%) discontinued due to disease progression. Two patients did not receive a complete infusion of BI 836826: one died of tumor lysis syndrome after a single dose of GemOx and did not receive any BI 836826, while a second had an interruption of the first BI 836826 infusion due to an IRR. This patient received 10 mg out of 25 mg BI 836826 and permanently discontinued trial drug.

### MTD and DLTs

BI 836826 dosing in combination with GemOx started at 25 mg and proceeded through 50 mg and 100 mg cohorts. Six patients were replaced and hence excluded from the MTD evaluation set. Two DLTs occurred during the MTD evaluation period (cycle 1), both grade 4 thrombocytopenia lasting > 7 days, affecting one of six evaluable patients (17%) in the 50 mg dosing cohort, and one of six evaluable patients (17%) in the 100 mg cohort. Due to a strategic decision by the sponsor, the trial was prematurely discontinued, and no further patients were enrolled after the 100 mg dose cohort. Consequently, the MTD of BI 836826 in combination with GemOx was not established.

### Safety

All patients experienced at least one AE during the on-treatment period of the trial, most commonly neutropenia (*n* = 16 [79%]), thrombocytopenia (*n* = 15 [71%]) and anemia (*n* = 14 [67%]). Sixteen patients (76%) experienced at least one AE deemed to be related to BI 836826 by the Investigator; the most frequent were (any grade/grade 3/4) neutropenia (52/38%), anemia (48/29%), thrombocytopenia (48/43%; Table 2). Eight patients (38%) experienced a treatment-related IRR, of which two (10%) were grade 3. Two patients (10%) had grade 4 neutropenia and a concomitant infection; both infections were of grade 2. One patient had grade 4 thrombocytopenia and concomitant bleeding, a single episode of grade 2 melena. The most common non-hematological AEs considered related to BI 836826 included pyrexia (*n* = 2 [9.5%]), asthenia (*n* = 2 [9.5%]), chills, cough, dizziness, nausea and vomiting (all *n* = 1 [4.8%]), which were either grade 1 or grade 2.

**Table 2:** AEs considered related to BI 836826 occurring in ≥ 2 patients

	Any-grade *n* (%)	Grade 3/4 *n* (%)
Any treatment-related AEs	16 (76.2)	12 (57.1)
Neutropenia	11 (52.4)	10 (47.6)
Anemia	10 (47.6)	6 (28.6)
Thrombocytopenia	10 (47.6)	9 (42.9)
Infusion-related reaction	8 (38.1)	2 (9.5)
Decreased white blood cell count	7 (33.3)	7 (33.3)
Increased AST	2 (9.5)	0
Increased GGT	2 (9.5)	1 (4.8)
Asthenia	2 (9.5)	0
Pyrexia	2 (9.5)	0
Decreased platelet count	1 (4.8)	1 (4.8)
Pancytopenia	1 (4.8)	1 (4.8)
Increased ALT	1 (4.8)	0
Chills	1 (4.8)	0
Cough	1 (4.8)	0
Dizziness	1 (4.8)	0
Hyperuricemia	1 (4.8)	0
Nausea	1 (4.8)	0
Vomiting	1 (4.8)	0

*ALT* alanine aminotransferase, *AST* aspartate aminotransferase, *GGT *gamma-glutamyl transferase

During the entire on-treatment period, seven patients experienced AEs that were consistent with the definition of a DLT. Thrombocytopenia (5 patients [24%]) was the only event reported for more than two patients.Six patients experienced a total of eight AEs leading to permanent discontinuation of last study medication: thrombocytopenia (two cases), neutropenia, *Aspergillus* infection, pneumonia, decreased white blood cell count, IRR, and tumor lysis syndrome (one case each).

Overall, 12 patients were reported with serious AEs (57%), of which only thrombocytopenia (4 patients [19%]) and IRR (3 patients [14%]) occurred in more than two patients. Three patients died during the study; however, no deaths were attributed to BI 836826. The case of tumor lysis syndrome was considered related to GemOx as the patient had not yet received BI 836926; the remaining two deaths were attributed to disease progression and *Aspergillus* infection.

### Efficacy

The objective response rate was 38%, including two patients (10%) with complete remission and six patients (29%) with partial remission (Table 3). Six patients (29%) experienced stable disease, and three patients (14%) had progressive disease. Four patients did not have a response assessment. Patients with baseline and at least one post-baseline disease assessment were evaluated for best change in SPD from baseline in lymph nodes, or organ or extra-lymphatic nodules based on PET/CT scan. The median best percentage change was − 31.3% (range –100 to 41%) for 15 patients with baseline lymph nodules and − 78% (range − 89% to − 28%) for five patients with extra-lymphatic disease nodules. In one patient with baseline liver nodules, change from baseline was − 38%, while two patients with baseline spleen nodules had changes of − 100% and − 45%.

**Table 3:** Best overall response in patients receiving BI 836826 plus GemOx

	BI 836826 dose			
Patients with response, *n* (%)	25 mg (*n* = 5)	50 mg (*n* = 8)	100 mg (*n* = 8)	Total (*n* = 21)
Complete remission	1 (20.0)	0	1 (12.5)	2 (9.5)
Partial remission	2 (40.0)	3 (37.5)	1 (12.5)	6 (28.6)
Stable disease	2 (40.0)	1 (12.5)	3 (37.5)	6 (28.6)
Progressive disease	0	2 (25.0)	1 (12.5)	3 (14.3)
Missing	0	2 (25.0)	2 (25.0)	4 (19.0)

*GemOx* gemcitabine 1000 mg/m^2^ and oxaliplatin 100 mg/m^2^

## Discussion

In this phase I, dose-finding study in patients with relapsed/refractory DLBCL, BI 836826 in combination with GemOx was generally well tolerated. The MTD was not established, as the trial was prematurely discontinued following the termination of the BI 836826 clinical development program. The MTD was not exceeded at the doses tested (50 and 100 mg given every 14 days).

Adverse events associated with BI 836826 administration were predominantly hematological, which is to be expected in this population, and in keeping with prior studies of BI 836826 in patients with B-cell malignancies [24, 30]. Grade 3/4 hematological AEs were more common in this study than with BI 836826 monotherapy [24, 30], most likely due to the additive effect of GemOx. One DLT of grade 4 thrombocytopenia lasting  > 7 days was recorded in each of the 50 mg and 100 mg dosing cohorts. As CD37 has been identified on megakaryocytes and platelets [31, 32], this may be the result of the direct action of BI 836826 on these cells, or may be related to the myelotoxic effects of gemcitabine and oxaliplatin [33, 34]. It is possible that the myelosuppressive effect of combination therapy was exacerbated by administering BI 836826 on day 8 of each cycle, corresponding with the nadir of hematological toxicity due to GemOx. Therefore, hematological toxicity could potentially be less severe with an alternative treatment schedule. However, the tested treatment schedule was based on modelling that evaluated different timings for the BI 836826 and GemOx administrations. The modelling showed that dosing BI 836826 after GemOx at the cytopenia nadir would actually shorten hematological toxicity compared to administering the drugs together, which was predicted to deepen and extend the cytopenia. Of note, the overall incidence of treatment-related IRRs (any-grade, 38%; grade 3/4, 10%) was lower than in a previous study of BI 836826 in patients with relapsed/refractory chronic lymphocytic leukemia (any-grade, 70%; grade 3/4, 8%) [24], and comparable to that seen in Caucasian patients with relapsed/refractory B-cell NHL (any-grade, 41%; grade 3/4, 8%) [30].

The overall response rate in this study was 38%. Few published data on the use of GemOx alone in DLBCL are available for comparison. In a retrospective study of GemOx with/without rituximab in 44 patients with relapsed/refractory aggressive lymphoma, overall response rate was 43%, including 30% CR [27], while an overall response rate of 61%, with 44% CR, was reported in a study of 49 patients with relapsed/refractory DLBCL who received rituximab plus GemOx [26]. However, considerable hematological toxicity was observed in both studies, with 73% of patients experiencing grade ≥ 3 neutropenia and 44% experiencing grade 3 thrombocytopenia in the latter study.

Although the clinical development of BI 836826 has been terminated for strategic reasons, the findings from this study suggest that CD37-based therapy for DLBCL can be safely combined with established chemotherapy regimens. A number of anti-CD37 therapies are currently being investigated for the treatment of B-cell NHL [20–22, 35–37], including radioimmunotherapy. Of note, a phase Ib study of the novel agent lutetium (177Lu)-lilotomabsatetraxetan (Betalutin®) in ASCT-ineligible patients with relapsed/refractory DLBCL is currently in progress (NCT02658968), and results from this and other ongoing clinical trials may provide new hope for DLBCL patients faced with limited options.

## Supplementary Information

ESM 1 (PDF 8 kb)

## Data Availability

The clinical study report (including appendices, but without line listings) and other clinical documents related to this study may be accessed on request. Prior to providing access, the documents and data will be examined, and, if necessary, redacted and de-identified to protect the personal data of study participants and personnel, and to respect the boundaries of the informed consent of the study participants. See https://trials.boehringer-ingelheim.com/data_sharing/sharing.html#accordion-1-2 for further details. Bona fide, qualified scientific and medical researchers may request access to de-identified, analyzable patient-level study data, together with documentation describing the structure and content of the datasets. Researchers should use https://clinicalstudydatarequest.com/ to request access to raw data from this study.
