# Supplementary material for: A phase Ib, open-label, dose-escalation trial of the anti-CD37 monoclonal antibody, BI 836826, in combination with gemcitabine and oxaliplatin in patients with relapsed/refractory diffuse large B-cell lymphoma
Source: Invest New Drugs. 2021 Feb 1;39(4):1028–35. doi: 10.1007/s10637-020-01054-6 (PMC8279964; doi:10.1007/s10637-020-01054-6)
Supplement: Supplementary file 1 — (PDF 8 kb) [file 10637_2020_1054_MOESM1_ESM.pdf]

Supplementary Table 1 Important protocol deviations

Protocol deviation	Patients*, n
Entrance criteria not met	
Efficacy-related entrance criteria not met	4
Patient did not have trial diagnosis or was not part of target population	1
Patient had a condition that may have caused additional risk from trial medication	1
Trial medication and randomization	
Delayed BI 836826 administration	3
Delayed GemOx administration	1
Concomitant medication	
Prohibited medication	1

*Three patients were identified with more than 1 protocol deviation: 1) did not have a CT scan with involvement of at least 1 bi-dimensional lesion/node >1.5 cm and delayed administration of BI 836826 in Cycle 1; 2) prior history of malignancy other than DLBCL and took prednisone for lymphoma, which was prohibited by the CTP; 3) HBV infection at the start of treatment, GemOx infusion on Day 2 instead of Day 1 of Cycle 1, and BI 836826 administration delayed in Cycle 1
